# Characteristics of surrounding rock damage and control technology of a facing-mining excavating roadway in north Shaanxi mining area

**DOI:** 10.1038/s41598-024-56295-9

**Published:** 2024-03-08

**Authors:** Li-Xin Zhang, Li Yi, Li Gang, Guang-Chao Liu, Ze-Hui Deng, Jia-Le Mi

**Affiliations:** School of Mining, Liaoning Technical University, Liaoning, 123032 China

**Keywords:** Facing-mining excavating, Numerical simulation, Damage characteristics, Surrounding rock control, Mineralogy, Petrology, Coal

## Abstract

In a coal mine in the northern region of Shaanxi Province, China, a facing-mining excavating roadway exists, which is intended to be retained for subsequent working face safety services. This paper investigates the deformation and damage characteristics of the surrounding rock in different stages using FLAC 3D numerical simulation, taking the facing-mining excavating roadway of this coal mine as the research context. At 20 m ahead of the working face, a discontinuous plastic zone appears in the surrounding rock of the roadway, a phenomenon attributed to the varying hardness of the lithologyand termed 'plastic zone jumping.' The numerical simulation results have been were verified using drill hole peeping. Real-time monitoring of the roadway's stability is conducted on-site, showing that the roadway is significantly affected by mining at the 50 m point ahead of the working face. Based on the numerical simulation and on-site monitoring results, the support strength was increased at 50 m from the working face along the roadway, and a new support scheme was adopted. In the lagging section of the roadway, where mining pressure is strongly evident, differentiated reinforcement using anchor rods, anchor ropes, and W steel belts has been employed, resulting in a satisfactory on-site effect.

## Introduction

Coal seams in the mining area of northern Shaanxi Province in China are generally characterized by shallow burial, large thickness, and relatively good geological conditions. These features enable a faster extraction rate of coal resources and the application of internationally advanced mining technologies, which significantly contribute to China's coal resource supply^1^. Factors such as the depth of coal seam burial, seam thickness, and the sequence of working face operations directly affect mining efficiency. Effectively addressing the issue of mining succession can greatly reduce the working cycle and enhance efficiency. Concurrently, controlling the stability of the roadway's surrounding rock under mining-induced stress is of paramount importance.

Facing-mining excavating is commonly adopted in practical engineering challenges. Scholars both domestically and internationally have extensively researched the stability of coal mine roadways subjected to facing-mining excavating activities, as well as the maintenance of the stability of the roadway's surrounding rock^2–7^. The anchoring process involves the mechanical interaction between the anchor and the surrounding rock. Currently, the more classical theories of anchor support include the arch theory, the loosening circle theory, the theory of the reinforcement of the strength of the surrounding rock and the theory of the new Austrian method of support, etc^8–13^. Additionally, numerous scholars have focused on reinforcing support schemes for return mining roadways under various conditions, including those influenced by mining activities and facing-mining excavating roadways^14–19^.

Di Xufeng et al.^20^ conducted research on the severe deformation and damage of the surrounding rock, as well as the susceptibility of the support components to failure, in the large mining highway of Mabao Coal Mine. They revealed the mechanism of deformation and damage and proposed the surrounding rock control technology of "roof and rib co-reinforcement". Wang Jie^21^ used the Yingming face of Xiaojiawa Coal Mine as the engineering context to study the relationship between perimeter rock deformation, stress, and support strength, and proposed appropriate control technology for the roadway's perimeter rock under mining influence. To address the issue of significant deformation of the surrounding rock and challenging support conditions in face-to-face mining with narrow coal pillars, Chen Xiaoxiang et al.^22^ researched the deformation law of the surrounding rock, the optimal size of the coal pillars, and the determination of corresponding support parameters during the face-to-face mining period. Kang Hui et al.^23^ examined the plastic zone deformation characteristics and causes during the entire lifecycle of underground paired-mining and paired-excavating roadways, with the Xiegou coal mine serving as the engineering background. Jiang Chongyang et al.^24^ analyzed the roadway under the influence of mining stress in Taoyuan Coal Mine, which experienced serious deformation and damage of the surrounding rock and presented difficulties in support, and identified the evolution law of roadway stress, deformation, and the plastic zone as the working face advanced. Using Limin Coal Mine as the engineering background, Yu Yang et al.^25^ utilized theoretical analysis, numerical calculations, and field tests to determine the optimal timing for excavating paired-mining and paired-digging roadways and proposed dynamic sub-area perimeter rock control technology based on the different stress environments of these roadways. Meichang Zhang et al.^26,27^ introduced an impact risk assessment method based on the BP neural network, evaluated its limitations and the degree of convergence, and also proposed a rockburst risk prediction method with outstanding field test results. Wang, X.H. et al.^28–30^ selected Tashan Coal Mine for their study to determine the deformation and damage mechanism of the haulage roadway near the close-range residual coal pillar and derived the implicit equation for the boundary of the plastic zone around the circular hole. Concurrently, they optimized the location of the return airway near the residual coal pillar for mining the close-range coal seam group in Tashan Mine.

To expedite the mining succession and enhance production efficiency, a coal mine in northern Shaanxi Province excavates the transportation roadway of the adjacent working face while the face is being mined out. This paper employs numerical simulation and on-site monitoring to investigate the deformation and damage characteristics of the surrounding rock of the facing-mining excavating roadway, thereby providing theoretical support for the roadway support, enhancing the stability of the perimeter rock, and ensuring the safety of the coal mine's production.

## Methods

### Engineering background

**Figure 1:**
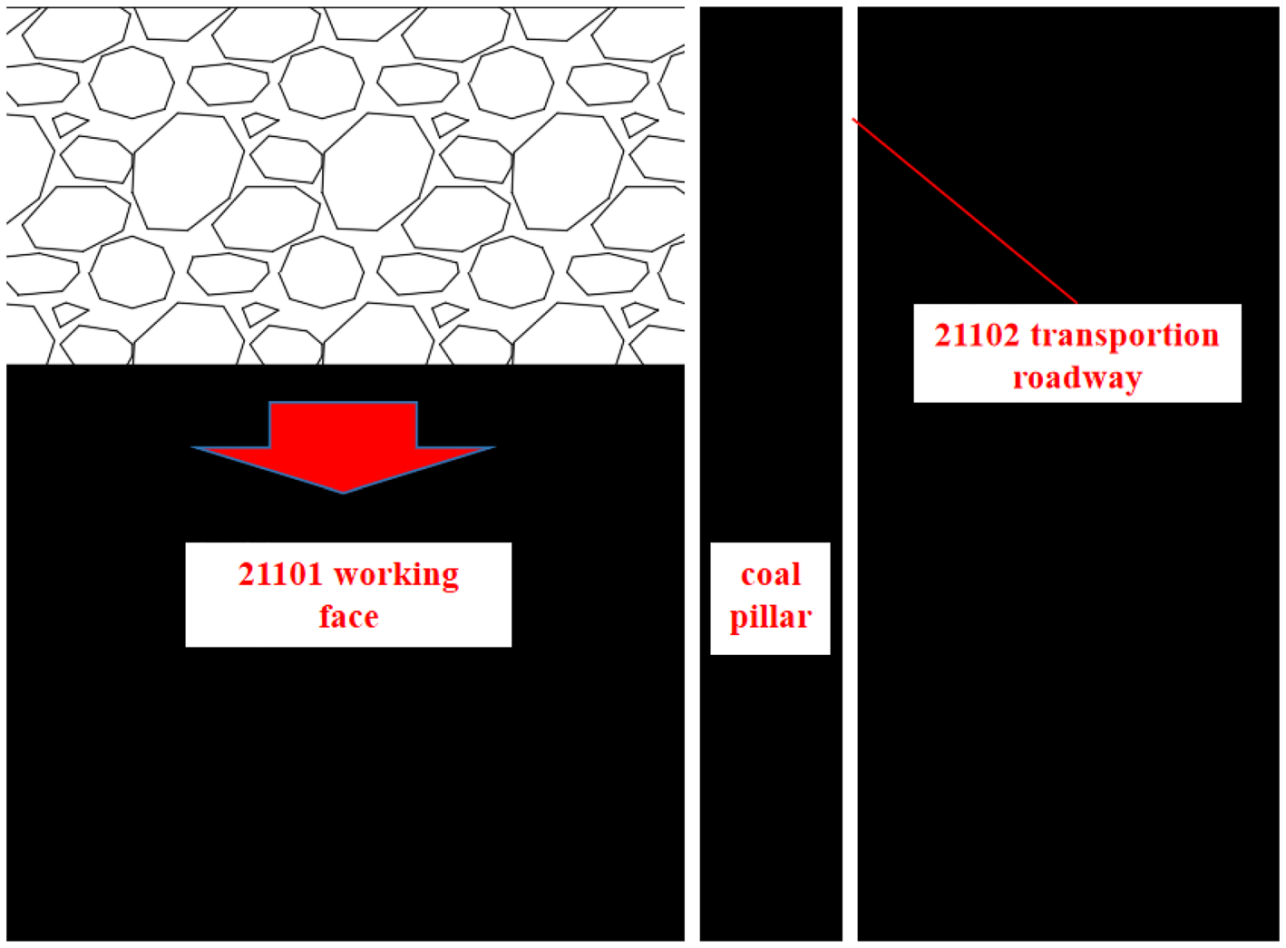
Position relationship diagram.

A coal mine is located in the northern area of Shaanxi Province, China. Currently, the main mining focus is the No. 2 coal seam, which has an average thickness of 3 m and an average depth of 260 m. This near-horizontal coal seam has a simple structure without gangue. The overlying strata are primarily siltstone and sandy mudstone, with local areas of fine-grained sandstone, while the underlying strata are mainly siltstone and sandy mudstone. The No. 2 seam's 21,101 working face is 140 m long, with an average spacing of 31.27 m from the No. 3 seam below it. The designed transportation and return roadways are both 4.8 m in width and 3 m in height, adopting the long-wall mining method to mine the full height in one pass. Roof management involves the complete caving method. The 21,101 working face is situated in the first mining area of the mine and is currently being mined in retreat. It is planned to design the 21,102 comprehensive mining face to the east of it, leaving a 20 m safety coal pillar, with the relative positional relationship depicted in Fig. 1.

To address the issue of mining succession and enhance production efficiency, the mine intends to construct the 21,102 transportation roadway while the 21,101 working face is being mined. Initially, the roadway was supported using the initial support program; at greater distances from the working face, the roadway experienced minimal deformation, and no phenomena such as gangue sheets occurred. However, as the working face and roadway advanced, the mining of the 21,101 working face gradually increased the deformation of the roadway, and dynamic disasters such as roof falls and rib spalling became apparent. To study the surrounding rock damage characteristics of the facing-mining excavating roadway, numerical simulation and on-site monitoring have been combined to conduct the study, proposing a corresponding supplementary support scheme. This scheme aims to control the deformation of the surrounding rock and maintain the stability of the 21,102 transportation roadway so that it can be retained and safely serve the 21,102 working face.

### Field monitoring program

A mining anchor dynamometer is used to observe changes in pressure on the anchor ropes. To monitor the condition of the anchor ropes and cables in the 21,102 transportation roadway at different distances from the 21,101 working face, an anchor dynamometer is employed for this purpose. To study the deformation patterns of the surrounding rock at various locations, continuous observations of the deformation of the top and bottom plates and the two rib sides of the roadway are made using a laser rangefinder.

Two anchor force gauges have been installed at a point 50 m ahead of the 21,102 transportation roadway working face, one on an anchor rod and the other on an anchor cable. Additionally, a surface displacement measuring point has been set up at the same section of the roadway.

Three borehole peeping measurement points are arranged at distances of 20 m ahead, at, and 30 m behind the 21,102 transportation roadway working face. Each point is located at the centerline of the roadway roof and is intended to observe the fracture development and damage tothe roof rock layer at different locations alongthe roadway.

### Numerical simulation

The stress surrounding a rock body will redistribute after being influenced by human engineering activities. During the mining process of the 21,101 working face, over-supporting pressure is produced in front of the coal body. When the excavation roadway, influenced by mining, is within the scope of this influence, the perimeter rock of the roadway will experience a superposition of stress states. The stress peak will increase significantly and when the stress exceeds the yield limit of the perimeter rock, plastic damage will occur. This may lead to phenomena such as rib spalling, top plate subsidence, and other disasters.

**Figure 2:**
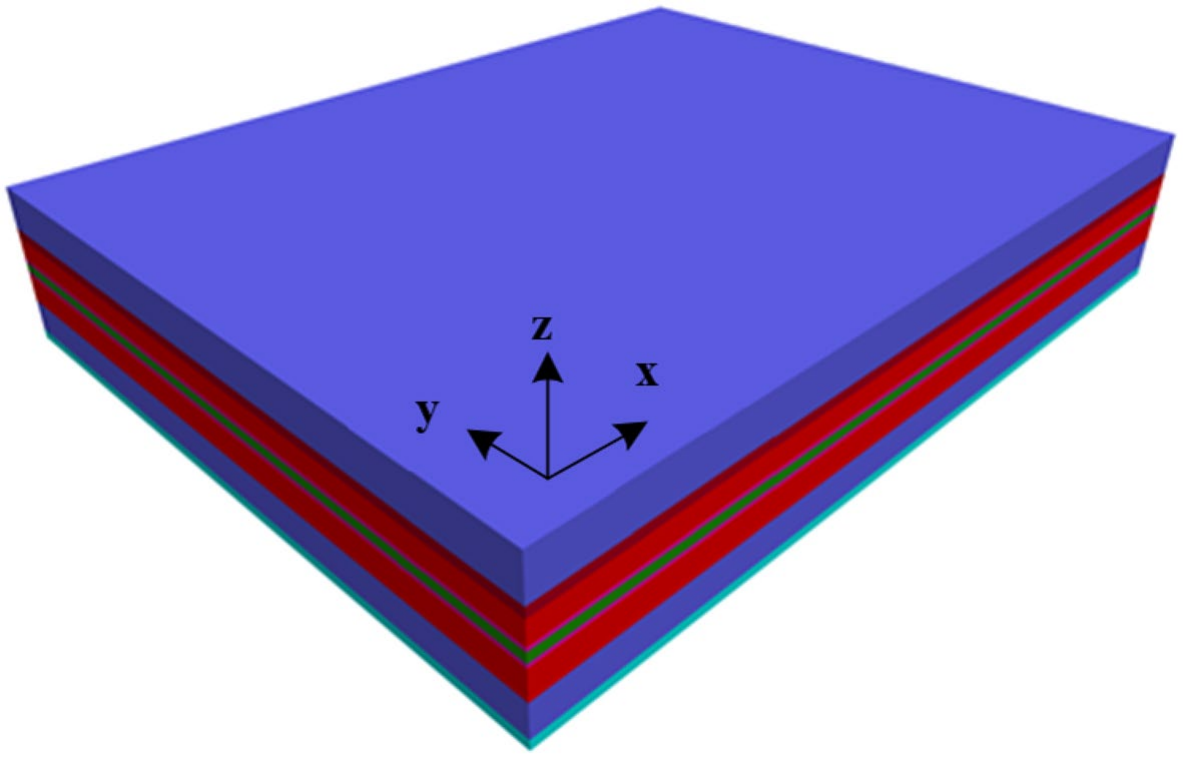
Diagram of the numerical model.

This paper focuses on a coal mine located in the northern area of Shaanxi Province, China, researching the surrounding rock damage characteristics of the mine’s facing-mining excavating roadway, specifically the 21,102 transportation roadway. This research will guide the control of the roadway’s perimeter rock. Taking into account the geological conditions of the mine and the actual situation on-site, a three-dimensional numerical model was established using FLAC3D numerical simulation software. As shown in Fig. 2, the model has dimensions of 220 m in length, 280 m in width, and 50 m in height, with displacement constraints applied to the bottom surface and surrounding interfaces of the model. A vertical stress of 5.5 MPa was applied to the top of the model, assuming a lateral pressure coefficient of 1. The overall model was defined as the Moore-Cullen intrinsic model. The grid around the location of the 21,102 transportation tunnel was locally refined to enhance the accuracy of the calculation results. The mechanical parameters of the rock used are listed in Table 1.

**Table 1:** Rock mechanics parameter table.

Parameters lithology	Density/(kg·m^3^)	Young/(GPa)	Cohesion/(MPa)	Friction/(°)	Tension(MPa)
31 coal	1500	3.8	0.9	21	0.35
Medium sandstone	2600	18.5	2.4	33	1.1
Siltstone	2550	21.5	2.18	30	0.8
21 coal	1500	3.8	0.9	21	0.35
Sandy mudstone	2580	18.9	3	30	0.95
Fine-grained sandstone	2650	22.8	2.5	32	0.84

## Results

### Roadway surrounding rock damage characteristics

**Figure 3:**
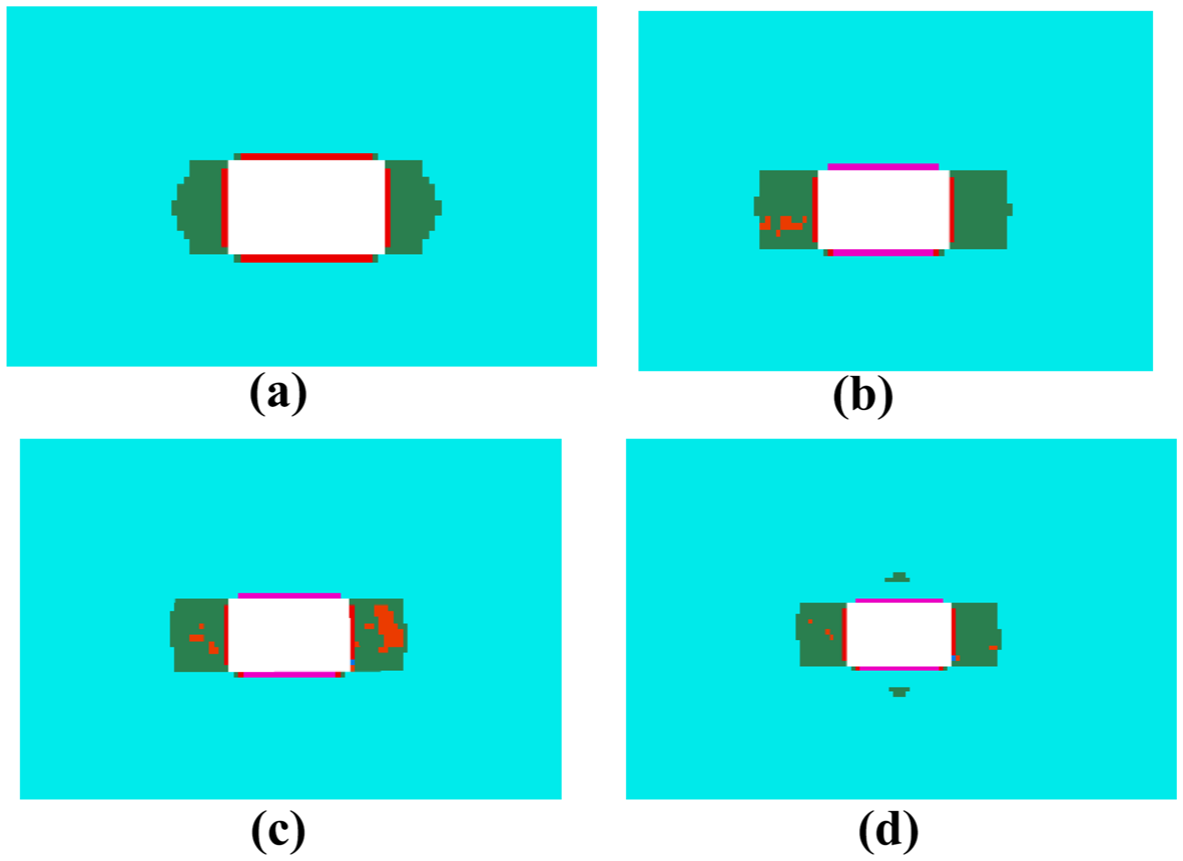
Distribution of plastic zones in the roadway at different locations of the ahead face.

Figure 3 illustrates the distribution of the plastic zone at different locations of the 21,102 transportation roadway relative to the 21,101 working face. From (a), it is observed that when the roadway is 120 m ahead of the working face, it is not affected by the superimposed pressure from the working face, and the plastic zone is symmetrically distributed in the roadway. The depth of the top and bottom plastic zones is relatively small, at 0.23 m, while the depth of the two ribs is larger, reaching 1.58 m. From (b), as the working face advances to within 100 m of the roadway, the extent of the top and bottom plate plastic zones remains unchanged, but the plastic zones in the ribs continue to develop. The maximum depth of the plastic zone increases to 2.35 m. When the roadway is 80 m ahead of the working face, the overall plastic zone of the roadway does not undergo significant changes; the increment in the depth of the plastic zones in the ribs is only 0.05 m, as shown in (c). As the roadway continues to be excavated, depicted in (d), when it is 20 m ahead of the working face, the ribs' plastic zones show no significant expansion. The top and bottom plates of the roadway exhibit discontinuous plastic zones. Starting from the existing plastic zones and extending a vertical distance of 0.75 m, a new plastic zone begins to develop. At this point, the maximum depth of the top plastic zone reaches 1.49 m, and the maximum depth of the bottom plate plastic zone reaches 1.47 m. This phenomenon is attributed to the varying hardness of the lithology, which causes the plastic zones to penetrate in jumps^31–34^.

**Figure 4:**
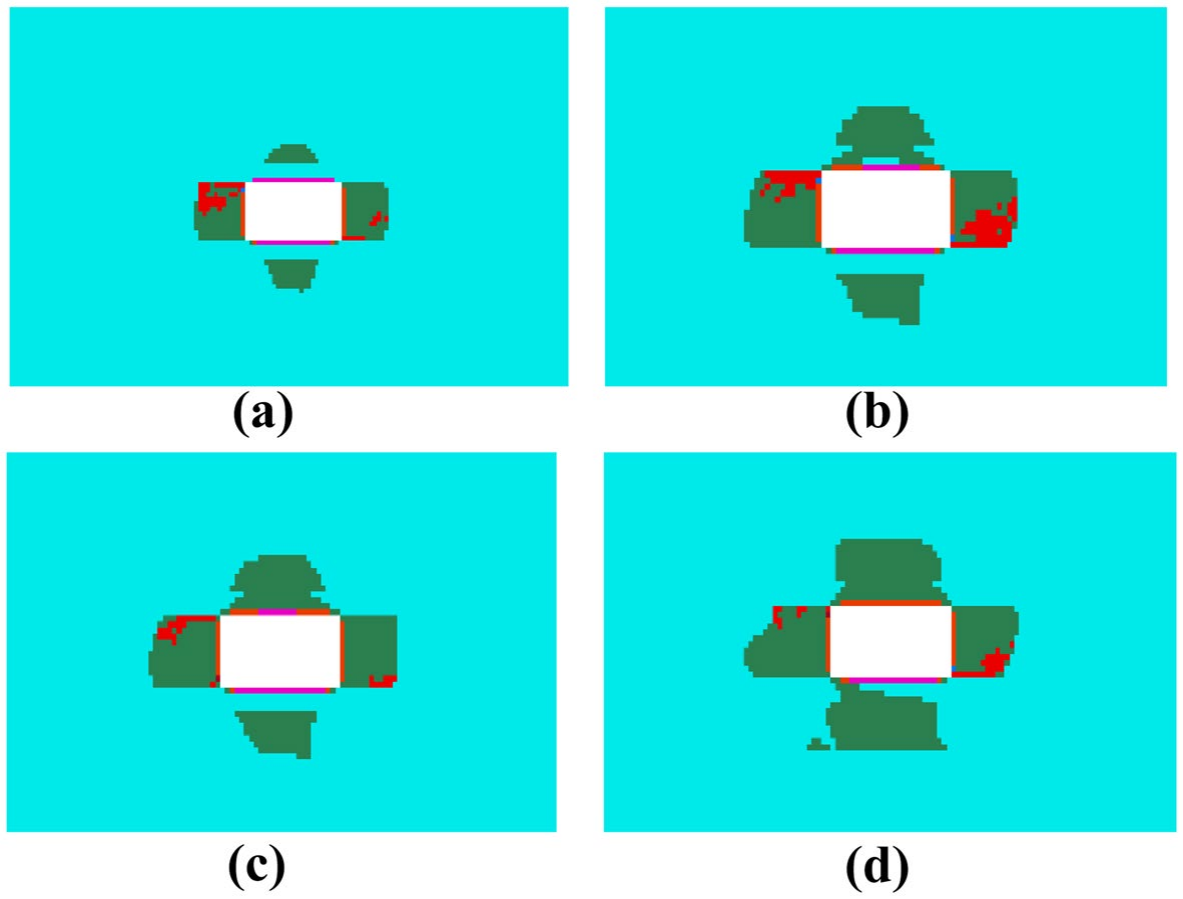
Distribution of plastic zones in the roadway at different locations of the lagging face.

As shown in Fig. 4, the mapping of the plastic zone distribution at different locations of the 21,102 transportation roadway lagging behind the 21,101 working face is presented. As the working face advances, the roadway enters the stage of lagging behind the working face. From (a), it is observed that when the roadway is directly at the working face (0 m lag), the discontinuous plastic zones at the center of the roadway's top and bottom plates continue to expand. The maximum depth of the plastic zone at the top plate reaches 1.98 m, while the maximum depth of the plastic zone at the bottom plate reaches 2.48 m. The sizes of the plastic zones in the ribs of the roadway also increase, with the maximum depth reaching 2.58 m. From (b), when the roadway is 20 m behind the working face, the plastic zones of the top plate begin to connect gradually. However, the overall size of the plastic zone does not change significantly. The plastic zone of the bottom plate remains disconnected, and the non-continuous plastic zone continues to develop downwards, with the maximum depth increasing to 2.72 m. When the roadway lags 60 m behind the working face, the new and old plastic zones of the top plate have completely connected, and the plastic zone begins to expand laterally, though the depth remains unchanged. The size of the bottom plate and the two plastic zones of the roadway have slightly increased. At a 100 m lag, as shown in (d), the plastic zone of the roadway's bottom plate gradually becomes connected and begins to extend to both sides, with the maximum size of the plastic zone reaching 3 m. The development of the plastic zone at the roadway's top plate is pronounced, with the maximum depth of damage reaching 2.73 m. The plastic zones in the ribs of the roadway display obvious asymmetrical characteristics, with the maximum depth of the plastic zone reaching 3.58 m. The plastic zones at the roadway's top plate and ribs are prominent, with the maximum depth of the plastic zone reaching 3.58 m.

### Surrounding rock deformation law

**Figure 5:**
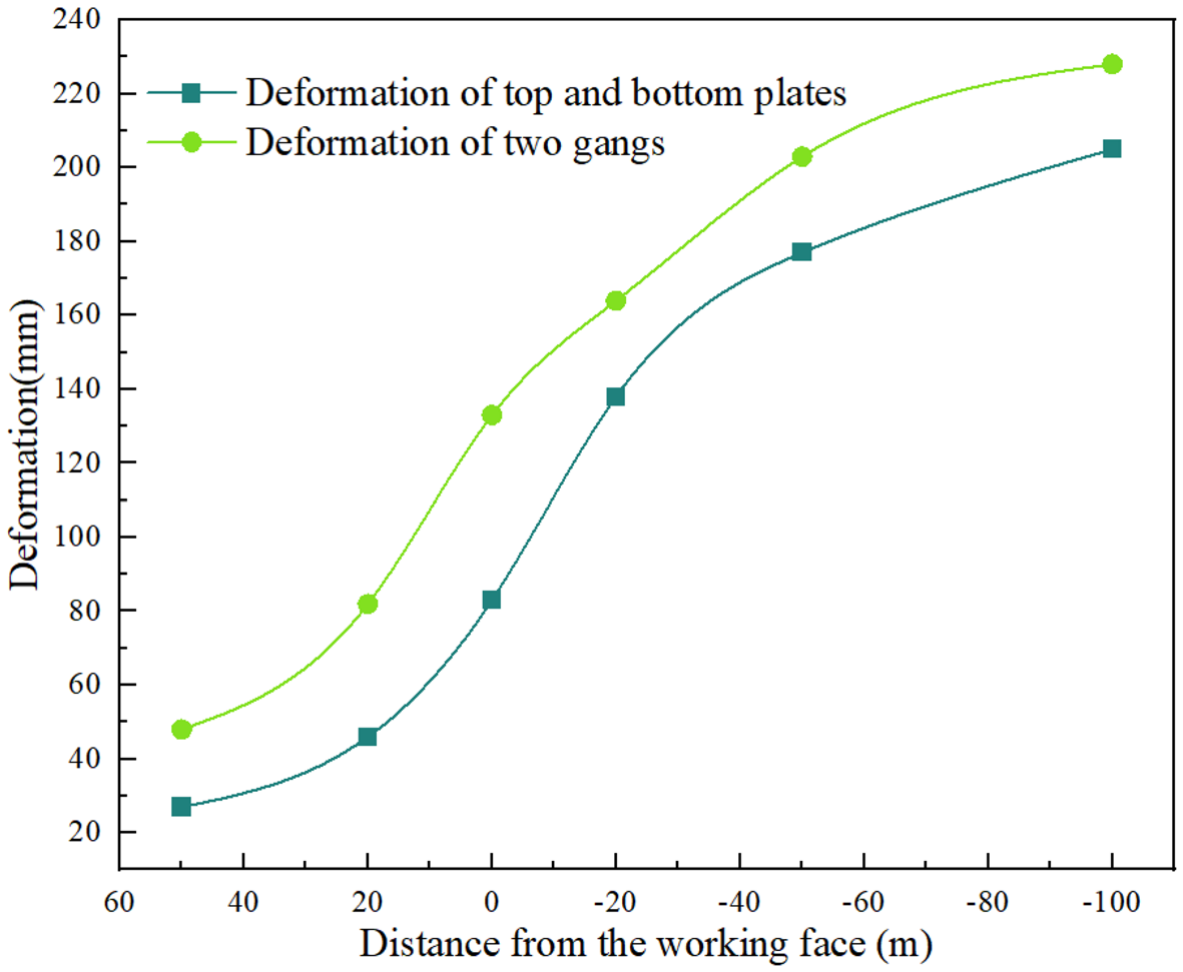
Deformation of the roadway at different positions from the working face.

The deformation of the top and bottom plates and the two ribs of the roadway at different positions from the working face is depicted in Fig. 5. The light green and blue lines represent the deformation change curves of the two ribs and the top and bottom plates of the roadway, respectively. As the relative distance between the 21,102 transportation roadway and the 21,101 working face decreases, the influence of the working face mining on the roadway becomes greater. Consequently, the deformation of the top and bottom plates, as well as the two ribs, also increases. The deformation of the roadway's top and bottom plates and two ribs at a distance of 50 m from the working face is 27 mm and 48 mm, respectively. The deformation of the top and bottom plates of the roadway begins to accelerate when at 0 m from the working face, and at − 100 m from the working face, the maximum deformation reaches 205 mm. The overall deformation trend of the two ribs of the roadway is similar to that of the top and bottom plates, with a significant increase at 20 m from the working face. At -100 m from the working face, their maximum deformation reaches 228 mm. It can be seen that the deformation of the roadway changes significantly under the influence of mining. To maintain the stability of the surrounding rock of the roadway and control the deformation of the surrounding rock, it is necessary to reinforce the roadway support.

### Support body working condition test

**Figure 6:**
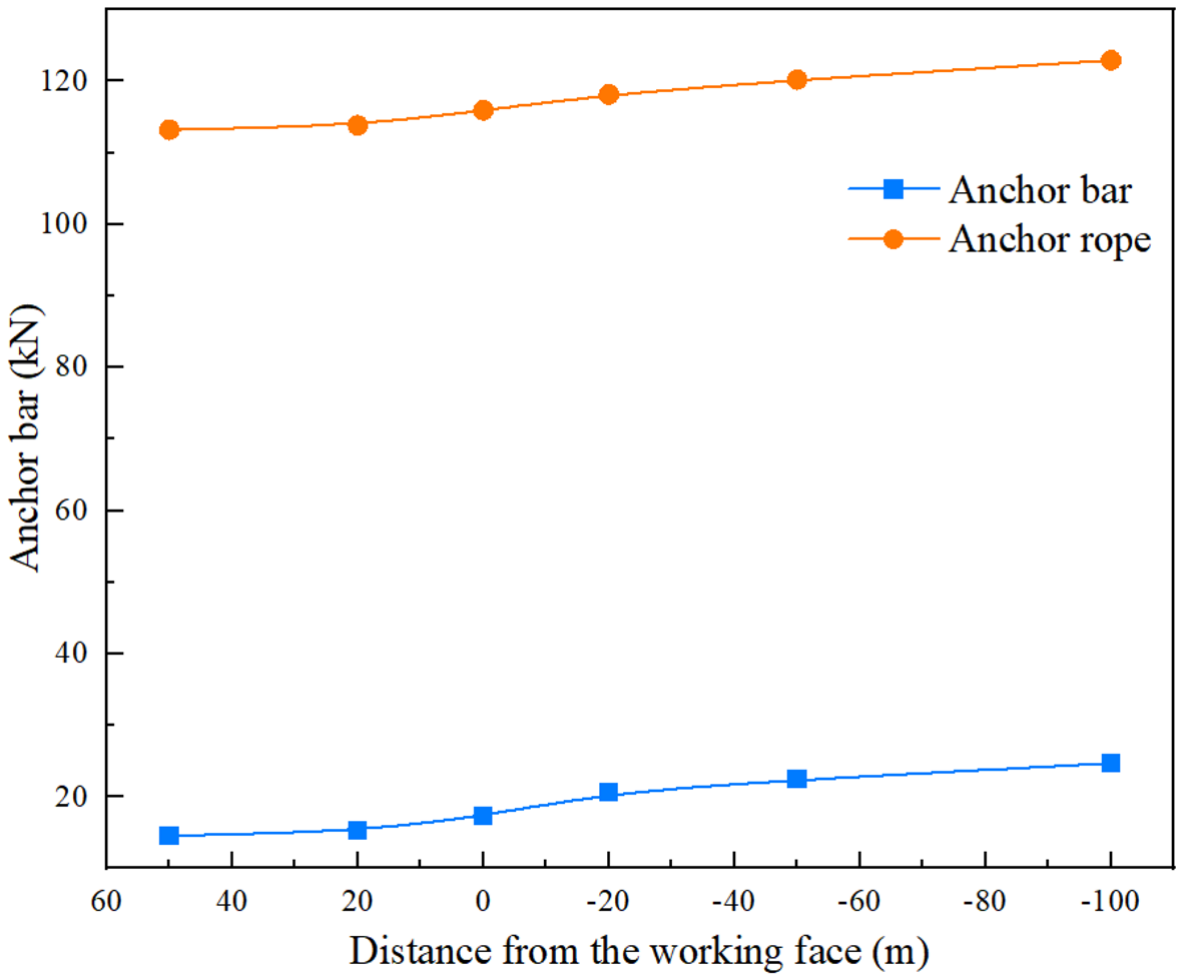
Anchor bar and anchor cable force at different positions from the working face.

Figure 6 shows the force curve of the anchor rods and cables at different locations of the roadway from the working face. The blue and orange curves represent the force variation curves of the anchor rods and anchor cables, respectively. It can be seen that the closer the roadway is to the working face, the greater the force exerted on the anchor rods. Initially, the force on the anchor cable is relatively stable, but after the superimposed perturbation of the working face mining, the force on the anchor cable increases. Corresponding to the numerical simulation results, the force on the anchor cable begins to increase substantially when a discontinuous plastic zone appears on the roof plate of the roadway. The larger the size of the plastic zone, the greater the increase in pressure on the anchor rods.

### Surrounding rock fissure development test

**Figure 7:**
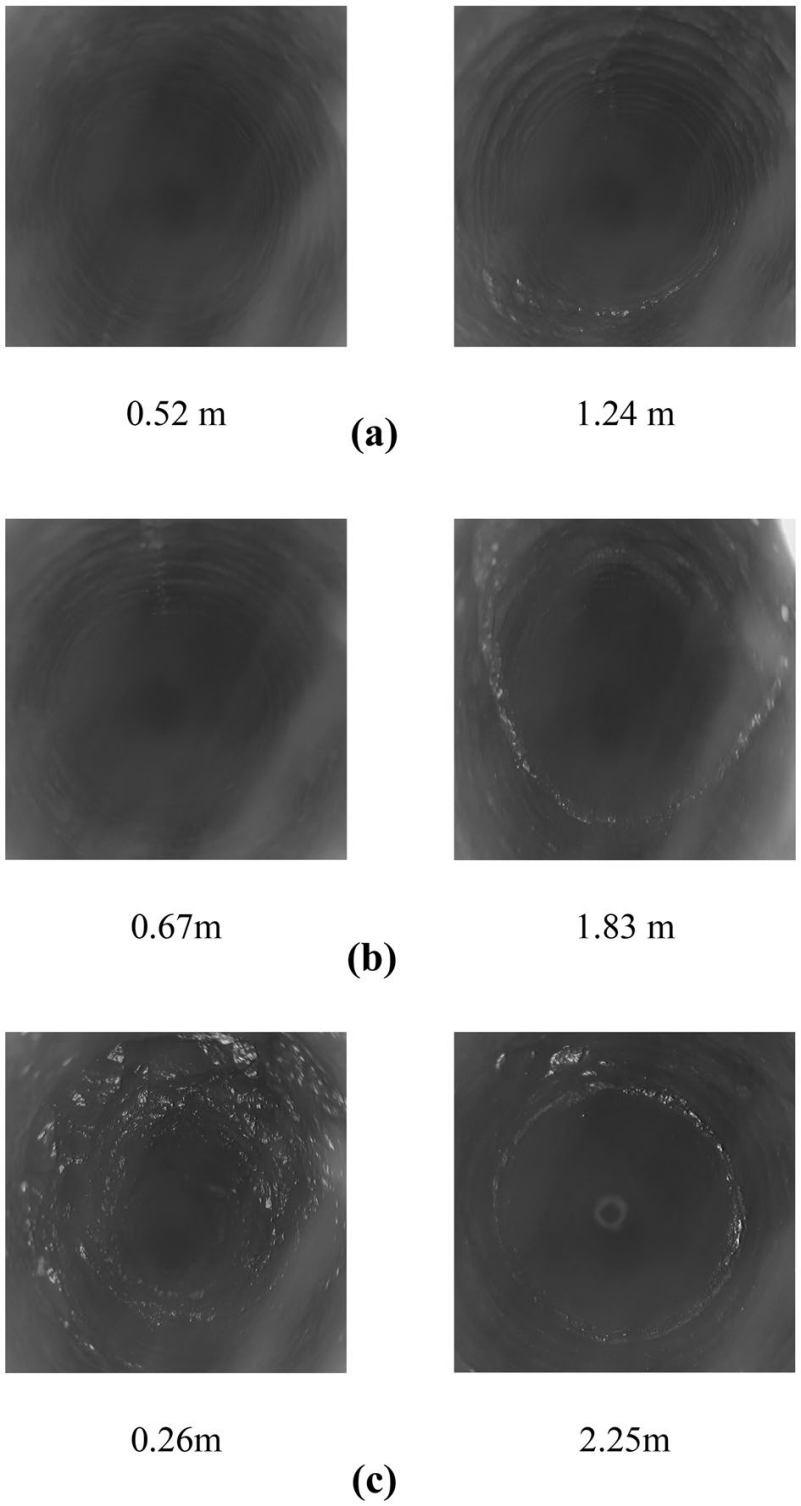
Fracture development of the top rock layer at different locations.

Based on the on-site drilling and peeping measurement points, the fissure development and damage of the roof rock layer at different locations of the 21,102 transportation roadway were detected. As shown in Fig. 7, at 20 m ahead of the working face, the rock wall at 0.52 m is relatively smooth, with no fissure development. At 1.24 m, transverse cracks appear in the rock wall, accompanied by other small cracks. The rock layer begins to show damage. At the 0 m position of the lagging working face, no damage is seen at 0.67 m of the top rock layer. However, at 1.83 m, the damage is more severe, with a large diagonal ring-shaped fissure approximately 0.03 m long. At the position where the working face lags by 30 m, the top rock layer has significant fissure development; from 0.26 m onwards, the rock layer is extensively damaged. Even at 2.25 m, a ring-shaped fissure still exists, accompanied by further fissure development. Overall, after lagging behind the working face, the roadway suffers greater damage to the top rock strata. Especially in the shallow positions, the top rock layer transitions from a more complete to a plastic damage state under the effect of mining disturbance.

## Discussion

**Figure 8:**
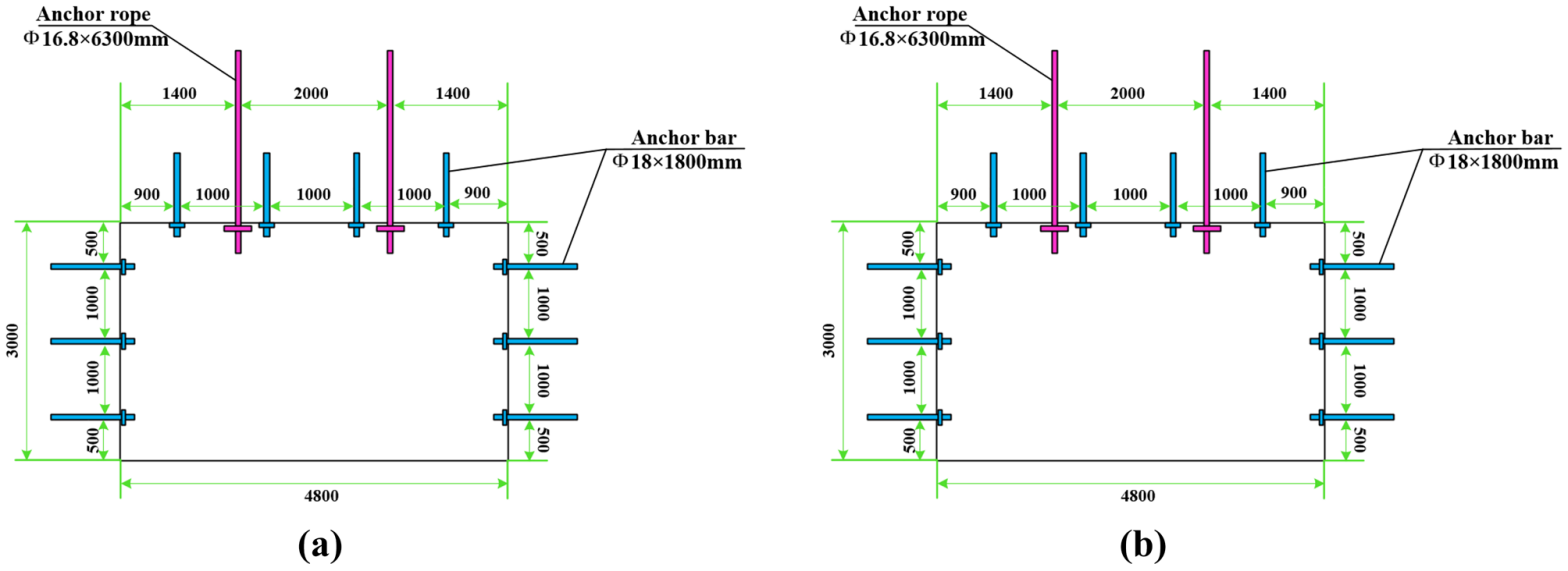
Schematic diagram of the different stages of support plan of the roadway.

From the results of the numerical simulation and on-site monitoring, it is evident that the 21,102 transportation roadway is significantly affected by the mining operations at the 21,101 working face. This activity leads to the deformation of the surrounding rock of the roadway, and the forces on the anchor ropes are increasing. Therefore, to preserve the stability of the surrounding rock of the roadway, allowing it to serve subsequent working faces more effectively, changes to the support scheme were initiated 50 m ahead of the working face, along with an increase in support intensity. Figure 8 displays the original support plan for the 21,102 transportation roadway alongside the revised support plan. The original support anchors were φ18 × 1800 mm with a row spacing of 1000 × 1000 mm, and the anchor cables were φ16.8 × 6300 mm with a row spacing of 2000 × 2000 mm. When the roadway is 50 m away from the working face, the original support scheme is insufficient to ensure the stability of the surrounding rock of the roadway. Consequently, a new support scheme is implemented. The new support scheme utilizes φ18 × 2300 mm anchor rods, adds an anchor rod to the rib of the roadway, and reduces the row spacing to 800 × 900 mm. The spacing of anchor rods at the top of the roadway remains unchanged, but the row spacing is adjusted to 900 mm. The anchor cables are upgraded to φ21.6 × 6300 mm, with an additional anchor cable added to the top of the roadway, and the row spacing is changed to 1200 × 1500 mm. It is also necessary to differentiate and reinforce the sections of the roadway lagging behind where the original support scheme was used, particularly where strong mining pressure is evident. Moreover, the lagging sections of the roadway that initially employed the original support plan need differentiation and reinforcement with φ18 × 2300 mm anchor rods and a W steel belt to prevent dynamic disasters, such as roof falls and rib spalling, due to mining influence, thereby ensuring the stability of the surrounding rock.

## Conclusions

(1) The discontinuous plastic zone of the roof plate begins to manifest at the 20 m point in the excavating roadway facing the mining at the working face, due to the influence of the mining movement. (2) Field tests indicate that the pressure and surface displacement of the roadway's anchor ropes gradually increase as the working face approaches. The roadway 50 m ahead of the working face experiences a more pronounced impact from mining.

(3) To maintain the stability of the roadway, the support strength is increased starting 50 m from the working face. Additionally, the section of the roadway that lags behind employs a combination of anchor rods and W steel belts for differentiated supplementary support, which has proven effective on-site.

## Data Availability

All data and materials are the property of the authors. Corresponding authors may provide raw data upon reasonable request.
